# Population dynamics of *Neisseria gonorrhoeae *in Shanghai, China: a comparative study

**DOI:** 10.1186/1471-2334-10-13

**Published:** 2010-01-21

**Authors:** Loubna Tazi, Marcos Pérez-Losada, Weiming Gu, Yang Yang, Lin Xue, Keith A Crandall, Raphael P Viscidi

**Affiliations:** 1 Division of Epidemiology and Disease Control, University of Texas, Health Science Center at Houston, School of Public Health, Brownsville Regional Campus, Brownsville, TX, USA; 2 Department of Biology, Brigham Young University, Provo, UT, USA; 3 CIBIO, Centro de Investigação em Biodiversidade e Recursos Genéticos, Universidade do Porto, Campus Agrário de Vairão, Vairão, Portugal; 4 Shanghai Skin Disease and STD Hospital, Shanghai, PR China; 5 Department of Pediatrics, Johns Hopkins University School of Medicine, Baltimore, MD, USA

## Abstract

**Background:**

Gonorrhea is a major sexually transmitted disease (STD) in many countries worldwide. The emergence of fluoroquinolone resistance has complicated efforts to control and treat this disease. We report the first study of the evolutionary processes acting on transmission dynamics of a resistant gonococcal population from Shanghai, China. We compare these findings with our previous study of the evolution of a fluoroquinolone sensitive gonococcal population from Baltimore, MD.

**Methods:**

Ninety six gonococcal samples were collected from male patients in Shanghai, China. All samples were fluoroquinolone resistant. Seven MLST housekeeping genes, two fluoroquinolone resistance genes (*gyrA *and *parC*) and the *porB *gene were sequenced and subjected to population genetic and evolutionary analyses. We estimated genetic diversity, recombination, growth, and selective pressure. The evolutionary history and population dynamics of the Shanghai population were also inferred and compared with that observed in a fluoroquinolone sensitive gonococcal population from Baltimore.

**Results:**

For both populations, mutation plays a larger role than recombination in the evolution of the *porB *gene, whereas the latter seems to be the main force driving the evolution of housekeeping and fluoroquinolone resistance genes. In both populations there was evidence for positively selected sites in all genes analyzed. The phylogenetic analyses showed no temporal clustering in the Shanghai gonococcal population, nor did we detect shared allelic profiles between the Shanghai and the Baltimore populations. Past population dynamics of gonococcal strains from Shanghai showed a rising relative effective population size (Ne) in MLST genes with a declining relative Ne for *gyrA *and *parC*, whereas among sensitive strains from Baltimore we previously observed concordance among these genes. In both Shanghai and Baltimore, the past population dynamics of gonococcal strains tracked changes in the prevalence of gonorrhea.

**Conclusions:**

Our study illustrates both similarities and differences in the evolutionary processes acting on gonococcal populations in different geographic areas. An explanation of this pattern that may apply in China is the continued use of quinolone antibiotics despite widespread resistance. Population genetic analysis of gonococcal strains in conjunction with epidemiological surveillance may provide insights into the epidemic behavior of antibiotic resistant strains and help to design control measures.

## Background

Gonorrhea is caused by *Neisseria gonorrhoeae*, a gram-negative aerobic diplococcus, and remains a major sexually transmitted disease (STD) in many countries worldwide [1-4]. The high prevalence of *N. gonorrhoeae *in developing countries and in some subpopulations in developed countries is related to poor accessibility to diagnostic tests and appropriate treatments [1,5]. Furthermore, inappropriate use of antibiotics has led to the development of resistant strains, although the prevalence of drug resistance varies greatly among countries [6-11]. The emergence of resistance to antimicrobial agents in gonococci represents a major challenge for public health control strategies in both developing and developed countries [4,12]. In fact, gonorrhea has proven difficult to control and remains a prime example of the influence that social, behavioral, and demographic factors, as well as microbial factors, can have on the epidemiology of an infectious disease.

In China, gonorrhea represents one of the most common sexually transmitted diseases and the most frequently reported STD in this country [9]. A total of 169,715 cases of gonorrhea were reported to the national Center for STD Control, CDC, China, in 2005 [13]. Even though the incidence of gonorrhea in this country is still increasing, population genetic and molecular epidemiologic studies remain limited [6,9,14].

Understanding the patterns of genetic diversity in microbial populations represents an important step for public health control strategies [15,16]. In fact, the characterization of the sources of such heterogeneity in these populations is relevant for the design of targeted treatment strategies. Previous population genetic studies of *N. gonorrhoeae *pointed out the role of recombination, mutation and selective pressures as driving forces for the evolution of this pathogen [10,17-19].

In order to understand the evolutionary processes acting on *N. gonorrhoeae *transmission dynamics in a community with a high incidence of gonorrhea, such as Shanghai, China, we estimated genetic diversity, recombination, growth, and selective pressure for the *porB*, seven housekeeping genes that constitute an MLST (MultiLocus Sequence Typing) scheme, and fluoroquinolone resistance genes from 96 Shanghai gonococcal isolates. We also inferred the evolutionary history and population dynamics of this population over the last 40 years. Finally, we compared the demographics of *N. gonorrhoea*e in Shanghai to that observed in another high incidence area, Baltimore, MD, USA [19]. In Shanghai, during the period of our study, gonorrhea incidence was 75 to 107 new cases per 100,000 inhabitants [20], while in Baltimore it was reported as 180 new cases per 100,000 inhabitants in 2005 [21]. However, in contrast with the Shanghai population that has a prevalence of fluoroquinolone resistant gonococci of 98% [6], in Baltimore such strains are still rare [22,23]. This comparative study will therefore allow for inferring common patterns of evolution and transmission dynamics between a resistant gonococcal population from Shanghai and a fluoroquinolone sensitive gonococcal population isolated in Baltimore.

## Methods

### Isolate collection and genes

96 gonococcal isolates were obtained between February 2001 and October 2005 from male patients seen in clinics of the Shanghai Skin Disease and STD Hospital. The epidemiological information for these isolates is presented in Additional file 1, Table S1. All the isolates were fully resistant to fluoroquinolone antibiotics. Ethical approval for the study was obtained from the ethical committee of the Shanghai Skin Disease and STD Hospital. All patients participating in the study gave oral informed consent.

Genomic DNA was extracted using the QIAamp DNA Mini Kit (Qiagen), according to the manufacturer's recommendations. Partial regions of seven core housekeeping genes *fumC*, *gdh*, *glnA*, *gnd*, *pilA*, *pyrD*, and *serC *[18,24], partial regions of *gyrA *and *parC*, which encompass the fluoroquinolone resistance-determining regions, and a fragment of the *porB *gene (PIA and PIB) were amplified by PCR and sequenced as described previously [19].

### Genetic analyses

In order to increase phylogenetic and statistical signal, the seven housekeeping gene fragments and the two fluoroquinolone resistance gene fragments were separately concatenated into two sequences for each isolate, providing a total of 288 DNA sequences for analysis, including 96 housekeeping gene sequences, 96 fluoroquinolone resistance gene sequences, 24 PIA sequences and 72 PIB sequences. The sequences were first translated into amino-acids using the universal genetic code in MacClade 4.05 [25], and then aligned using MAFFT [26]. Amino-acid sequences were then backtranslated into DNA sequences and used for our genetic analyses. Unique sequences were deposited in GenBank under accession numbers EU796227 to EU796257 and EU796259 to EU796357.

Gene phylogenies were estimated using maximum likelihood analyses [27] with nodal support assessed via bootstrapping (1,000 pseudoreplicates) [28], as implemented in PHYML [29]. Gene phylogenies were also assessed using Bayesian methods [30] coupled with Markov Chain Monte Carlo (BMCMC) inference, as implemented in MrBayes v3.1.2 [31]. Model selection for these analyses followed the procedure outlined by Posada and Buckley [32], as implemented in ModelTest v3.6 [33] using the Akaike Information Criterion (AIC). The substitution model GTR+Γ+I was selected for the housekeeping genes and the fluoroquinolone resistance genes as the best-fit model of molecular evolution. The models of molecular evolution chosen for PIA and PIB groups were GTR+I and HKY+Γ+I, respectively. For the BMCMC techniques, two independent analyses were run each consisting of four chains. Each Markov chain started from a random tree and ran for 2.0 × 10^7 ^cycles, sampling every 1000^th ^generation. In order to confirm that our Bayesian analyses converged and mixed well, we monitored the likelihood scores and compared means and variances of all likelihood parameters and likelihood scores from the independent runs using the program Tracer v1.4 [34].

Evolutionary relationships among the different genes were also assessed using the method of statistical parsimony [35], as implemented in the software package TCS v1.21 [36]. TCS networks were constructed so that the solid squares (putative outgroups) and solid circles represent actual sequences derived from the strains analyzed. The size of the colored squares and circles is proportional to the number of sequences displaying the same genotype. Each open circle represents putative sequences in the evolutionary pathway. The solid lines on a network represent mutational connections among unique genotypes with at least a 95% degree of confidence, whereas the dashed lines represent a more tenuous connection (*P *< 0.95).

In addition to phylogenetic and network reconstruction approaches, the *F*_ST _fixation index, as implemented in ARLEQUIN v3.1 [37], was estimated to test for temporal structuring of the gonococcal population. *P *values below 0.05 were considered statistically significant.

Genetic diversity (*θ*), recombination rates (*r*), and growth rates (*g*) were estimated for each set of genes using the maximum likelihood coalescent approach implemented in LAMARC v2.0.2 [38]. Three independent runs were performed for each gene in order to assess the reproducibility of the different parameter estimates. Furthermore, an estimate of the relative contribution of recombination (*C*) to genetic diversity was obtained by multiplying *r *and *θ *[39].

The impact of natural selection was inferred by estimating the ratio of nonsynonymous to synonymous substitutions (*ω *= d_N_/d_S_) per site and per gene using the codon-based nested models M1 (neutral)/M2 (selection), and M7 (beta)/M8 (beta and *ω*), as implemented in the PAML package v4 [40]. The likelihood of evolution was therefore compared under models prohibiting (M1, M7) versus models allowing (M2, M8) positive selection. Models M2 (three parameters) and M8 (four parameters) extend M1 (one parameter) and M7 (two parameters) by adding a third class of residues with *ω *> 1. Model likelihood scores were compared using a Likelihood Ratio Test (LRT) to determine the best-fit model. The Bayes empirical Bayes approach was applied to identify the potential sites under diversifying selection as indicated by a posterior probability (p*P *≥ 0.95) [41]. The past population dynamics of *N. gonorrhoeae *in Shanghai were inferred using the Bayesian skyline plot model [42], as implemented in BEAST v1.4.6 [43]. BEAST assumes no recombination and we are not aware of any study testing the robustness of BEAST to violation of the no recombination assumption. However, preliminary simulations by our group indicate that BEAST is indeed robust to levels of recombination similar to those observed in the Shanghai population (Additional file 2, Figure S1). Nonetheless, before performing our analyses, we searched for recombinant isolates in our datasets using RDP3 [44]. All algorithms were considered and applied to estimate the number of recombination events and no recombinants were detected in our datasets, presumably due to the shortness of the sequence fragments. Log-normal relaxed clock evolutionary models were used for our analyses [45]. Lower and upper limits and mean of the substitution rates were obtained from an extensive *N. gonorrhoeae *dataset from Baltimore recently analyzed by our group [19]. The Bayesian Markov chain Monte Carlo (MCMC) outputs generated by BEAST were analyzed using Tracer v1.4 [34].

Recent evolutionary relationships among the housekeeping gene sequences were analyzed using the eBURST algorithm implemented at http://eburst.mlst.net/[46]. Unique alleles for each of the seven housekeeping genes were given a numerical identifier and isolates with identical allelic profiles were assigned the same sequence type (ST) number. Groups of STs were defined as a clonal complex if they shared at least five of seven alleles.

## Results

**Figure 1:**
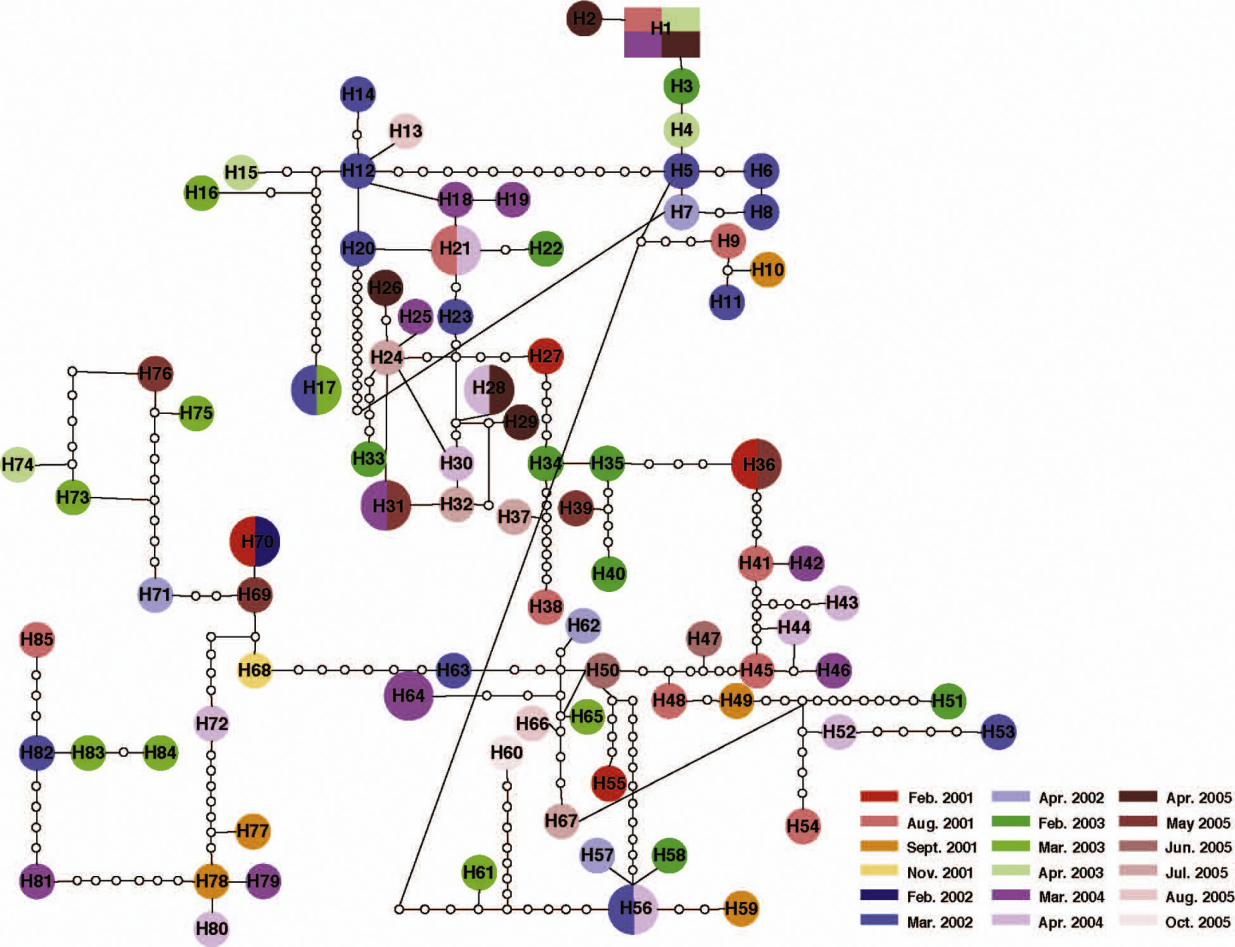
**Statistical parsimony network of concatenated housekeeping genes (*fumC*, *gdh*, *glnA*, *gnd*, *pilA*, *pyrD*, and *serC*)**. The solid squares (putative outgroups) and the solid circles represent actual sequences derived from the strains analyzed. The size of squares and circles is proportional to the number of sequences displaying the same genotype. The open circles represent putative sequences in the evolutionary pathway. The solid lines on a network represent mutational connections among unique genotypes with at least a 95% degree of confidence, whereas the dashed lines represent a more tenuous connection.

Among the 96 strains collected in Shanghai between 2001 and 2005, the phylogenies constructed by maximum likelihood and Bayesian methods did not show any clustering related to the date of isolation (data not shown). The evolutionary relationships assessed using network approaches also failed to reveal temporal clustering in this population, as illustrated by the network reconstruction for the housekeeping genes (Figure 1). The other genes showed also similar patterns in these analyses (Additional files 3, 4 and 5, Figures S2-S4). The quantitative analyses for detecting temporal structuring (*F*_ST _analyses) were also not significant for all year comparisons and for all genes analyzed, except for the fluoroquinolone resistance genes in which very few of these analyses (only 5 among the total 26 analyses for these genes) showed significant *P *values. However, after Benferroni correction, among these five significant associations, only one year comparison (2002 - 2005) remains significant (Additional file 6, Table S2). This finding suggests therefore no temporal genetic differentiation between the isolates from Shanghai.

The network reconstructions for all the genes indicated a high level of genetic diversity among the isolates, with the greatest diversity observed in the housekeeping genes. In fact, 85 different genotypes were present in the housekeeping genes, with almost 90% of the total genotypes as unique (Figure 1). The networks of the fluoroquinolone resistance genes and the *porB *PIA and PIB genes showed 37, 7, and 40 different genotypes, respectively (Additional files 3, 4 and 5, Figures S2-S4). The unique genotypes among the fluoroquinolone resistance genes and the *porB *PIA and PIB genes comprised about 52%, 72%, and 65%, respectively, of the total genotypes in the Shanghai population.

To further assess genetic differences among the strains collected in Shanghai, we estimated population genetic parameters, including genetic diversity (*θ*), recombination (*r *and *C*), and growth (*g*) using LAMARC v2.0.2 for the concatenated housekeeping genes, the concatenated fluoroquinolone resistance genes, and *porB *gene (PIA and PIB) (Table 1). We also estimated the population genetic parameters partitioning the genes by date of collection, with the exception of the PIA sequences due to the small size of this dataset (Table 1). The PIB sequences showed higher genetic diversity than the housekeeping sequences, the PIA sequences or the fluoroquinolone resistance sequences. Moreover, the ratio of the per-site rate of recombination to the per-site rate of mutation (*c*/*μ*) in the *porB *gene indicates that mutation plays a greater role compared to recombination in the evolution of this gene. On the other hand, the fluoroquinolone resistance genes showed the highest recombination rates in comparison to the other genes (Table 1). In fact, recombination appears to be the main driving force for the evolution of the housekeeping genes and the fluoroquinolone resistance genes. The genetic diversity and recombination rate estimates showed, in general, similar values, when considering the partitions by date of collection (Table 1). However, the fluoroquinolone resistance genes showed a higher genetic diversity for the samples isolated in 2005. The estimates of the population genetic parameters for the individual housekeeping and fluoroquinolone resistance genes, in general, supported the results from the analysis of the concatenated genes; however, for some genes the levels of genetic diversity and recombination were low (Additional file 7, Table S3). Overall, growth rates (*g*) showed high positive values (i.e., exponential growth) in all genes analyzed, except for PIA sequences where *g *showed a negative value (Table 1). This latter result may reflect a decrease in the expression of PIA alleles in the Shanghai population. Also, PIA included fewer sequences (24 isolates) than the housekeeping genes (96 isolates), the fluoroquinolone resistance genes (96 isolates) and the PIB genes (72 isolates), and *g *estimation seems to be very sensitive to sample size [38]. The *g *values were also positive for each time point and for all gene partitions.

**Table 1:** Population genetics estimates for the Shanghai gonococcal population.

	*N*_*s*_	*θ*	*r*(*c*/*μ*)	*C*	*g*
MLST genes	96	0.02 [0.017 to 0.023]	1.39 [1.21 to 1.58]	0.03 [0.02 to 0.04]	1,240 [1,052 to 1,420]

2001	19	0.006 [0.005 to 0.008]	1.45 [0.99 to 1.94]	0.009 [0.005 to 0.015]	1,032 [746 to 1,306]

2002	18	0.005 [0.004 to 0.007]	1.27 [0.62 to 1.72]	0.006 [0.002 to 0.012]	453 [286 to 605]

2003	20	0.005 [0.004 to 0.008]	1.6 [1.27 to 1.97]	0.008 [0.005 to 0.016]	815 [501 to 1,087]

2004	20	0.005 [0.004 to 0.006]	1.79 [1.37 to 2.3]	0.009 [0.005 to 0.014]	684 [463 to 890]

2005	19	0.006 [0.004 to 0.001]	1.58 [1.05 to 2.13]	0.009 [0.004 to 0.02]	654 [291 to 1,297]

*gyrA *and *parC*	96	0.015 [0.012 to 0.018]	4.97 [4.11 to 5.94]	0.07 [0.05 to 0.11]	2,147 [1,777 to 2,489]

2001	19	0.013 [0.007 to 0.024]	2.18 [1.23 to 3.22]	0.03 [0.009 to 0.08]	1,767 [980 to 2,573]

2002	18	0.007 [0.004 to 0.017]	3.56 [1.72 to 5.41]	0.02 [0.007 to 0.09]	2,643 [1,232 to 4,725]

2003	20	0.009 [0.006 to 0.019]	3.29 [2.12 to 5.57]	0.03 [0.01 to 0.11]	1,970 [1,280 to 5,629]

2004	20	0.009 [0.003 to 0.038]	1.31 [0.51 to 3.11]	0.012 [0.001 to 0.118]	914 [77 to 2,732]

2005	19	0.032 [0.007 to 0.067]	2.8 [1.73 to 5.78]	0.09 [0.012 to 0.39]	3,633 [912 to 5,142]

PIA	24	0.007 [0.004 to 0.016]	0.049 [0.002 to 0.23]	0.0003 [0.00001 to 0.004	(-56) [(-277) to 128]

PIB	72	0.047 [0.035 to 0.067]	0.51 [0.33 to 0.73]	0.024 [0.01 to 0.05]	39 [(-2) to 87]

2001	12	0.04 [0.018 to 0.094]	0.34 [0.16 to 0.63]	0.014 [0.003 to 0.06]	24 [(-44) to 97]

2002	14	0.04 [0.02 to 0.09]	0.3 [0.12 to 0.58]	0.012 [0.002 to 0.05]	26 [(-27) to 86]

2003	16	0.032 [0.017 to 0.066]	0.24 [0.1 to 0.47]	0.008 [0.002 to 0.03]	10 [(-47) to 67]

2004	12	0.044 [0.018 to 0.121]	0.18 [0.03 to 0.51]	0.008 [0.0005 to 0.06]	47 [(-44) to 147]

2005	18	0.032 [0.017 to 0.061]	0.29 [0.13 to 0.54]	0.009 [0.002 to 0.03]	18 [(-44) to 80]

Estimates of genetic diversity (*θ*), recombination (*r *and *C*), and growth (*g*) of seven concatenated housekeeping genes, two concatenated fluoroquinolone resistance genes, and the *porB *gene (PIA and PIB) for the entire Shanghai gonococcal population and partitions by year of isolation. LAMARC's confidence intervals (5% and 95%) around the estimates of each parameter are given between brackets. *N*_*S *_= Number of sequences (isolates).

We also tested for the extent of natural selection in the Shanghai gonococcal population and found that all models that allow for positively selected sites (M2 and M8) suggest the existence of these sites in all the genes analyzed (Table 2). In fact, all models indicate the presence of a proportion of sites (1.5% - 14%) under very strong positive selection (10.5 - 75.3). The likelihood ratio tests comparing models M1 vs M2 and models M7 vs M8 were all significant (*P *< 0.001). The Bayesian approach identified 14 positively selected sites (p*P *≥ 0.95) in the housekeeping genes under model M2 and 15 positively selected sites under model M8. All of the 14 sites detected under model M2 were included in the 15 sites found by model M8. Within the fluoroquinolone resistance genes, models M2 and M8 both detected the same six positively selected sites. Among these sites under positive selection, two sites were detected in *gyrA *gene whereas the four other sites were detected in *parC *gene. In *gyrA *gene, the two positively selected sites correspond to the sites known to confer resistance to quinolones (Ser-91 → Phe mutation and Asp-95 → Ala mutation). On the other hand, among the four positively selected sites detected in *parC *gene, only two sites were already described as sites conferring resistance to quinolones (sites Ser-87 and Glu-91 with no mutations at these residues) [6]. For this study, quinolone-resistant *N. gonorrhoeae *isolates from Shanghai presented double mutations of *gyrA *gene (Ser-91 → Phe and Asp-95 → Ala) combined with no mutation of *parC *gene, which corresponds to one of the most predominant patterns (Pattern P3) already described in another resistant gonococcal population from Shanghai [6]. Four positively selected sites were found in PIA sequences under models M2 and M8; and in the PIB sequences, 10 and 12 positively selected sites were detected under models M2 and M8, respectively. The 10 sites found under model M2 were all included in the 12 sites detected under model M8.

**Table 2:** Log-likelihood values and parameter estimates for the Shanghai gonococcal population.

	***InL***_**M1**_	***InL***_**M2**_	***ω***_**M2**_	***p***_**M2**_	***n***_**M2**_	***InL***_**M7**_	***InL***_**M8**_	***ω***_**M8**_	***p***_**M8**_	***n***_**M8**_
MLST genes	-7113.9	-7026.6	10.5	0.015	14	-7128.3	-7026.6	10.5	0.015	15

*gyrA *and *parC*	-1504.4	-1439.6	75.3	0.023	6	-1506.5	-1439.6	75.1	0.023	6

PIA	-749.9	-741.8	28.3	0.14	4	-749.9	-741.8	28.5	0.14	4

PIB	-1426.4	-1378	12.7	0.08	10	-1427	-1378.4	13.9	0.07	12

Log-likelihood values and parameter estimates (*ω*, *p*, *n*) of seven concatenated housekeeping genes, two concatenated fluoroquinolone resistance genes, and the *porB *gene (PIA and PIB). *lnL *= Likelihood values; *ω *= acceptance rate per site (*ω*_M2 _and *ω*_M8_); *p *= proportion of sites under diversifying selection (*p*_M2 _and *p*_M8_); *n *= number of positively selected sites with a posterior probability > 0.95 (*n*_M2 _and *n*_M8_). All comparisons between nested models (M1 vs M2, and M7 vs M8) were significant.

**Figure 2:**
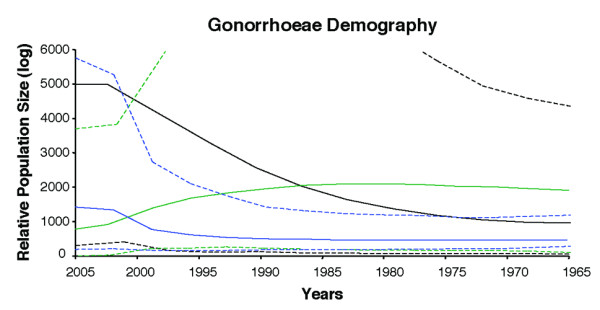
***N. gonorrhoeae *population dynamics in Shanghai over the past 40 years**. Relative population size was estimated from seven housekeeping genes (black lines), two fluoroquinolone resistance genes (green lines), and *porB *gene (blue lines) with the solid lines showing the mean estimates and the dashed lines showing the 95% highest posterior density (HPD) limits.

We inferred the past population dynamics of *N. gonorrhoeae *in Shanghai using the BEAST program. The population dynamics of the gonococcal population in Shanghai over the past 40 years inferred from the housekeeping genes showed that the relative effective population size (Ne) increased dramatically and steadily from the early 1980s until 2002 and then leveled off (Figure 2). The inference made from the *porB *gene showed that relative Ne was level until the late 1990s and subsequently increased sharply from 1997 until 2002 and more slowly since that time. The confidence limits around the estimate are wide and it cannot be excluded that the rise began in the early 1990s and has been much steeper than the estimate derived from the mean value (Figure 2). In contrast, the fluoroquinolone resistance genes showed a different scenario for the population dynamics of *N. gonorrhoeae *with stable to slightly increasing relative Ne until 1985 followed by a sharp decline reaching its minimum size in 2005.

**Figure 3:**
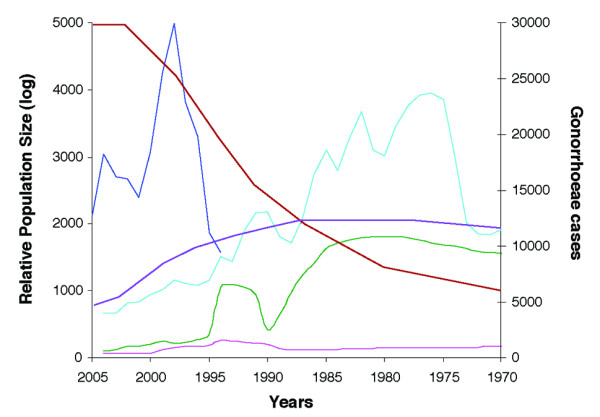
***N. gonorrhoeae *population dynamics in Shanghai and Baltimore over the past 35 years**. Comparison of reported cases and relative population size of *N. gonorrhoeae *in Shanghai and Baltimore over the past 35 years (from 1970 to 2005). The light blue line shows the number of reported cases of *N. gonorrhoeae *in Baltimore (1970 - 2004), and the dark blue line shows the reported cases of *N. gonorrhoeae *in Shanghai (1994 - 2005). Relative population size was estimated from the housekeeping genes (brown line: Shanghai; green line: Baltimore), and the fluoroquinolone resistance genes (purple line: Shanghai; pink line: Baltimore). Only the mean estimates are shown.

We also compared the Shanghai gonococcal population with that from a previous study we conducted using isolates from Baltimore, MD. The comparison between the gonococcal populations isolated in the two geographical areas showed different population dynamics over the past 35 years in each of these settings (Figure 3). Over the past 25 years (from 1980 until 2005), the relative Ne inferred from the housekeeping genes declined dramatically in Baltimore except for a transient rise and fall between 1990-1995, whereas it increased in Shanghai. A different population dynamics was also observed for the fluoroquinolone resistance genes in the Baltimore and Shanghai populations over the past 25 years (Figure 3). Relative Ne was stable in Baltimore, apart from a slight rise and fall, which paralleled the changes seen for the housekeeping genes in the 1990-1995 period, while in Shanghai, relative Ne declined between 1985 and the present, as noted above. In both populations the changes in relative Ne inferred from the housekeeping genes tracked trends in the total number of reported cases of *N. gonorrhoeae*. Thus, in Baltimore the general decline in cases from 1975 until the present corresponded to a decline in relative Ne, with the exception noted above for the period 1990-1995. Although reporting for Shanghai is incomplete, the dramatic increase in reported cases between 1994 and 1998 was reflected in a steep rise in relative Ne during this time period (Figure 3).

**Figure 4:**
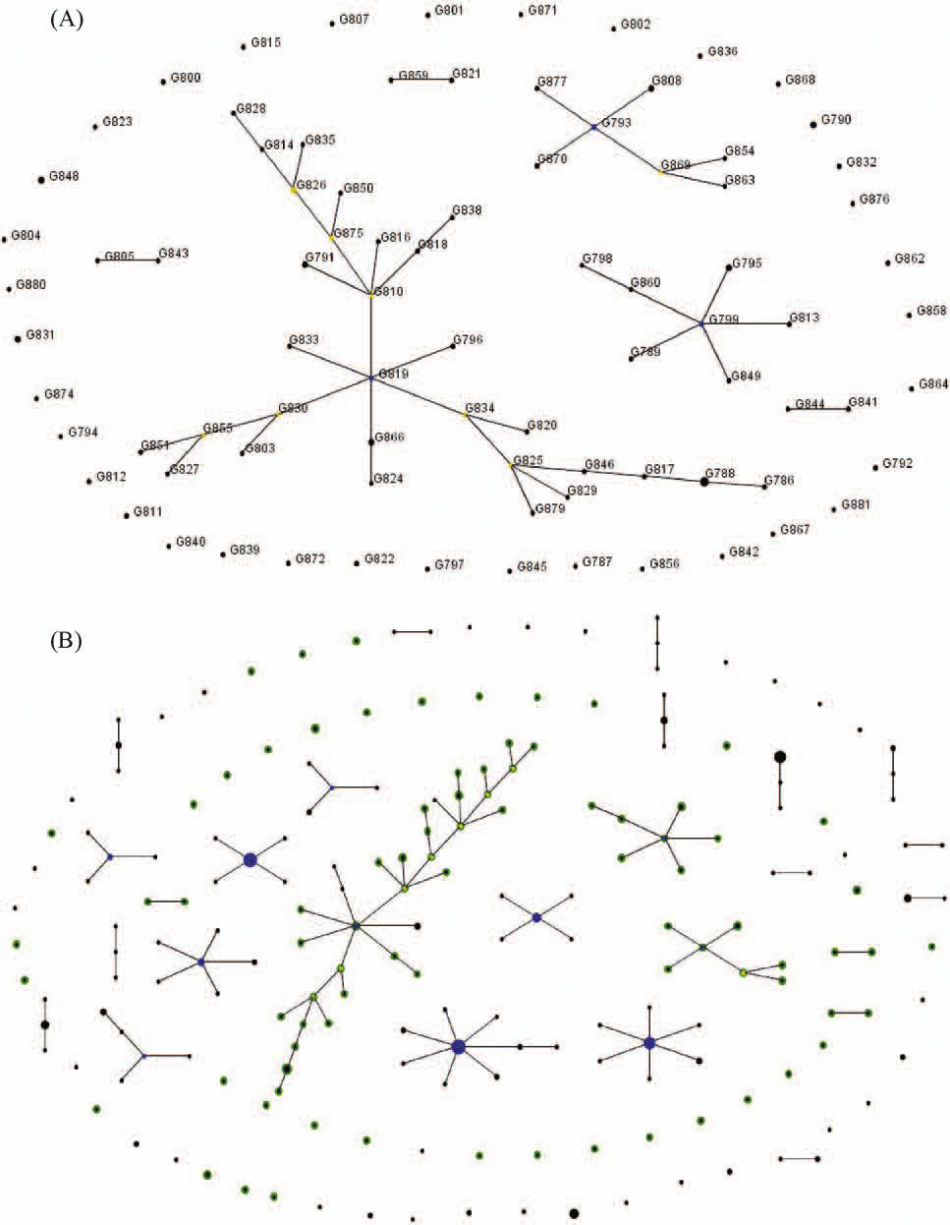
**eBURST diagram displaying the recent evolutionary relationships among MLST sequences in the gonococcal population**. (A) eBURST diagram displaying the relatedness of 96 Shanghai gonococcal isolates. The size of each circle is proportional to the number of isolates with the same ST. Clusters of linked isolates correspond to clonal complexes. Primary founders in the cluster are shown in blue, and the subgroup founders are shown in yellow. (B) eBURST diagram displaying the comparison between the Baltimore and the Shanghai populations. The size of each circle is proportional to the number of isolates with the same ST. Clusters of linked isolates correspond to clonal complexes. The STs found only in the Shanghai population are shown in green, and the STs found only in the Baltimore population are shown in black. If STs are found in both populations, they are shown in cyan.

Finally, we analyzed recent evolutionary relationships among the housekeeping gene sequences in the Shanghai gonococcal population using the eBURST algorithm. The allelic profiles of the 96 isolates were classified into two groups with 76 STs in group 1 and three STs in group 2, in addition to six other unlinked STs. In the eBURST diagram, the three major clonal complexes did not include the isolates collected during the same period of time; and the primary founders in two clonal complexes (G793 and G799) were isolated in 2005 (Figure 4-A). The founder ST (G819) for the largest clonal complex was isolated in 2002. For this dataset, the level of recombination is moderate and the nodes in the largest clonal complex represent therefore true lines of descent. The eBURST analysis supports the findings of the phylogenetic and *F*_ST _analyses that there is no temporal genetic differentiation between the isolates from the Shanghai population. We also compared the Shanghai and Baltimore populations using this algorithm in order to detect STs common to both populations. The STs for each population are colored differently in the diagram. No STs shared by both populations were detected (Figure 4-B). Additionally, *F*_ST _analysis revealed significant differences between both populations (*P *< 0.001).

## Discussion

Fluoroquinolones have been widely used for the treatment of gonorrhea since the 1980s because of their strong antibacterial efficiency in addition to their lower cost and the convenience of oral administration. However, resistance to these drugs emerged quickly in many countries [47-51]. In China, the rate of fluoroquinolone resistance is very high, reaching even 100% in some areas[52]. However, these drugs are still used to treat gonorrhea in this country because all health providers do not follow official recommendations for antibiotics administration and antibiotics are widely available without a prescription. Despite the rapid spread of resistant gonococci in this region, information on population genetics and epidemiology of *N. gonorrhoeae *in China remain limited [6,9,14]. Our study represents the first study to focus on understanding the evolutionary processes acting on the transmission dynamics of resistant gonococci in Shanghai.

Our analysis showed that the evolutionary processes acting on the fluoroquinolone resistant gonococcal population from Shanghai shared many features with what we previously described for a fluoroquinolone sensitive population from Baltimore [17-19]. For both populations, mutation plays a larger role than recombination in the evolution of the *porB *gene, whereas the latter seems to be the main force driving the evolution of the housekeeping and fluoroquinolone resistance genes [18,19]. While it is clear that recombinational hotspots occur in genomes (e.g., [53]), the causes of such hotspots remain a mystery [54]. The differences observed in this study seem to be related to differences in selection regimes for the different genes in question. The housekeeping genes are under purifying selection, which leads to a larger number of synonymous relative to nonsynonymous substitutions, and would select against rearrangements due to recombination that would cause fitness declines. For these genes, recombination would therefore play a larger role in creating genetic diversity by shuffling synonymous mutations into novel allelic combinations. The *porB *gene and fluoroquinolone resistance genes, on the other hand, are more likely to be impacted by diversifying selection and directional selection, respectively. The *porB *gene undergoes diversifying selection due to its interaction with the immune system to escape immune detection, and we can then expect, as observed in our analyses, a high impact of mutation for generating diversity in this gene. Interestingly, such selection pressure seems also to lead to a dominant role for recombination to shuffle existing variation into novel combinations. The impact of recombination should therefore be incorporated into vaccine strategies that are centered around the *porB *gene [55,56]. The fluoroquinolone resistance genes, on the other hand, are exposed to directional selection to acquire substitution changes associated with drug resistance. Apparently, novel combinations produced through recombination aid also directional selection. In fact, this evolutionary process appears to be the dominant force relative to mutation for genes under both directional and diversifying selection. Indeed, the evolution of these genes is analogous to the *env *and *protease *genes (i.e., diversifying selection to avoid immune detection and evolution of drug resistance, respectively) in HIV where recombination has a major impact [57]. This interpretation is also supported by the selection analysis that showed a much higher level in the proportion of positively selected sites in the fluoroquinolone resistance genes relative to the housekeeping genes and an increase in selection in the *porB *gene as well (Table 2). Indeed, the *porB *gene shows an 8-fold increase in the proportion of sites under diversifying selection relative to the housekeeping genes.

Additionally, in both populations, estimates of growth rate (*g*) showed high positive values for all genes with the exception of PIA sequences. A likely explanation for the negative value for the PIA sequences is the small number of sequences included in the analysis since *g *estimation seemed to be very sensitive to sample size, as illustrated by the large confidence interval for this estimate in these sequences. However, it cannot be excluded that there is a decrease in expression of PIA alleles in the gonococcal population as the same observation was made in the Baltimore population. The phylogenetic analyses and the network reconstructions showed no temporal clustering among the Shanghai strains, similar to our previous findings for the gonococcal population from Baltimore [18]. The population genetic analyses of the Shanghai isolates showed similar levels of genetic diversity for time partition, providing further evidence for the absence of temporal clustering. One exception was that isolates collected in 2005 showed a higher level of genetic diversity for the fluoroquinolone resistance genes than isolates collected in the four previous years. Contrary to the Shanghai population, the gonococcal population from Baltimore showed evidence for temporal clustering in the Baltimore population based on the *F*_ST _estimates. However, it is important to note that the sampling interval (1 year) and time span (5 years) of the Shanghai study population were less than those of the Baltimore study, where isolates were collected every fifth year over a 15 year time period. This difference in sampling strategy may have limited our ability to detect temporal clustering. However, it is important to emphasize that the sample size of the Shanghai population is appropriate and adequate for generating population genetic estimates [58].

Changes in relative Ne over time represent a valuable tool for inferring the current and past epidemiology of infectious diseases. Our analysis of population dynamics of *N. gonorrhoeae *over the past 40 years in Shanghai, inferred from the housekeeping genes, showed that relative Ne has been increasing since the early 1980s with a leveling off from 2002 until 2005. The inference made from the *porB *gene was similar to that observed with the housekeeping genes. In contrast, the fluoroquinolone resistance genes showed a declining relative Ne since 1985. The appearance of antibiotic resistant strains would be predicted to cause a decrease in relative Ne since a limited number of strains with resistance mutations would have a selective advantage. This expectation was born out by the BEAST analysis of the fluoroquinolone resistance genes. Additionally, a decrease in relative Ne estimated from the housekeeping and *porB *genes would also be predicted, due to a selective sweep. However, the opposite pattern was observed in this population: relative Ne estimated from these genes increased (Figure 2). The most likely explanation for this finding is that fluoroquinolone antibiotics continue to be used widely in China and their continued use led to rapid diversification as the resistance genes spread throughout the gonococcal population. The evolutionary pattern of a rising relative Ne in housekeeping genes in the presence of a declining relative Ne for resistance genes may have therefore broad application in clinical practice as an indication that antibiotics are being misused and the need for better strategies for managing the resistance. However, we cannot exclude the possibility that social and epidemiological factors also contributed to the increase in relative Ne inferred from the housekeeping and *porB *genes. The rise in incidence of gonorrhea in late 1990s has been mainly attributed to the influx of people into Shanghai and to economic development attracting more construction workers from the countryside to that area. This movement of people may have resulted in the importation of new strains into the city, fueling the increase in relative Ne. Conversely, the recent leveling off or decline in relative Ne observed in our analysis may be related to a concerted effort by the public health services to prevent and treat new *N. gonorrhoeae *cases.

The comparative analysis of past population dynamics of gonococcal populations from Shanghai and Baltimore revealed significant differences between these populations (Figure 3). Relative Ne inferred from housekeeping genes declined dramatically in Baltimore over the past 25 years, while relative Ne in Shanghai increased sharply. In both geographic areas, the changes in relative Ne appeared to largely track changes in the total number of reported cases of *N. gonorrhoeae*. The historical patterns of prevalence of gonorrhea influence therefore deeply its population dynamics. This finding is indeed reassuring that the BEAST analysis is robust despite the recombination level detected in this population. We observed similar trends in relative Ne from housekeeping and fluoroquinolone resistance genes in our analysis of Baltimore strains, compared to the results from the Shanghai gonococcal strains. This is not surprising since fluoroquinolone resistance was not reported in Baltimore until 2005 [19,22], and thus *gyrA *and *parC *genes were under the same selective pressure as the housekeeping genes.

The eBURST analysis showed also that the Shanghai population is very different and unique, compared to the Baltimore population. No ST was shared between the two populations. The lack of identical STs shared by these two populations provides evidence for geographic structuring of gonococcal populations, as previously shown in analyses using *porB *sequences of strains from different geographic regions [17]. However, phylogenetic analysis of the combined Shanghai and Baltimore MLST dataset did not reveal segregation of gonococcal isolates between the two cities (data not shown). The eBURST methodology appears, therefore, to have greater sensitivity for identifying recent clonal populations, or at least applies a much more stringent definition for such populations. This approach emphasizes also the continuous emergence of resistant strains in Shanghai and the urgent need for better control and prevention strategies in this area.

## Conclusions

In comparison to our previous study of sensitive gonococcal strains in Baltimore, this population genetic analysis of quinolone resistant strains revealed differences that may have microbiological and clinical implications. The more dominant role of recombination compared to mutation in the evolution of resistant strains raises the possibility that horizontal gene transfer is an important mechanism for the acquisition of resistance.

Future studies should investigate potential environmental sources for resistance conferring determinants. Although quinolone resistance genes function as housekeeping genes and among sensitive strains the population dynamics of these genes paralleled that of the MLST genes, in the resistant population in Shanghai we found a rising relative Ne in MLST genes in the presence of a declining relative Ne for resistant strains, indicating continuous diversification of resistant strains rather than spread of a clonal resistant population. We speculate that one explanation for this pattern is continued use of an ineffective antibiotic with acquisition of resistance by strains with novel genotypes. In our previous study of quinolone resistant strains from Israel [10], we saw a pattern characteristic of the spread of an epidemic strain with low levels of diversity at all tested loci. Given these different evolutionary pictures, monitoring the population dynamics of *N. gonorrhoeae *strains at MLST and antibiotic resistance loci could assist in the design of control measures that focus, for example, on core transmitters in the latter case or on poorly informed providers in the former scenario. In this study of gonorrhea in Shanghai as well as our previous study of gonorrhea in Baltimore, we noted that the pas t population dynamics of gonocooci mirrored the historical pattern of the prevalence of gonorrhea. Future prospective studies are needed to see if changes in population dynamics have predictive value and thus would assist in the design of control measures.

## Competing interests

The authors declare that they have no competing interests.

## Authors' contributions

LT participated in the design of the study, carried out the genetic analyses and drafted the manuscript. MP-L participated in the design of the study, assisted with past population dynamics analyses and helped to draft the manuscript. WG supervised the collection of gonococcal strains. YY carried out sample preparation. LX generated nucleotide sequence data. KAC participated in the conception and design of the study, obtained funding for the study, and helped to draft the manuscript. RPV participated in the conception and design of the study, obtained funding for the study, supervised the generation of nucleotide sequence data and helped to draft the manuscript. All authors read and approved the final manuscript.

## Pre-publication history

The pre-publication history for this paper can be accessed here:

http://www.biomedcentral.com/1471-2334/10/13/prepub

## Supplementary Material

Additional file 1 **Epidemiological information for the isolates under study**. All isolates were collected from patients seen in clinics of the Shanghai Skin Diseases Hospital. Click here for file

Additional file 2 **BEAST simulations for population size (A) and TMRCA (B)**. Simulations performed under different values of recombination per site (X axis). 6,000 datasets were simulated in Recodon [59] under conditions reflecting average estimates for selection (*ω*), recombination (*r*) and substitution rate (*μ*) parameters in gonorrhea studies for the housekeeping, fluoroquinolone resistant, and *porB *genes. Simulated data were then analyzed in BEAST to assess the impact of recombination alone (*ω *= 1) and combined with adaptive selection (*ω *> 1) and purifying selection (*ω *< 1). Click here for file

Additional file 3 **Statistical parsimony network of concatenated fluoroquinolone resistance genes (*parC *and *gyrA*)**. The solid squares (putative outgroups) and the solid circles represent actual sequences derived from the strains analyzed. The size of squares and circles is proportional to the number of sequences displaying the same genotype. The open circles represent putative sequences in the evolutionary pathway. The solid lines on a network represent mutational connections among unique genotypes with at least a 95% degree of confidence, whereas the dashed lines represent a more tenuous connection. Click here for file

Additional file 4 **Statistical parsimony network of *porB *gene (PIA)**. The solid squares (putative outgroups) and the solid circles represent actual sequences derived from the strains analyzed. The size of squares and circles is proportional to the number of sequences displaying the same genotype. The open circles represent putative sequences in the evolutionary pathway. The solid lines on a network represent mutational connections among unique genotypes with at least a 95% degree of confidence, whereas the dashed lines represent a more tenuous connection. Click here for file

Additional file 5 **Statistical parsimony network of *porB *gene (PIB)**. The solid squares (putative outgroups) and the solid circles represent actual sequences derived from the strains analyzed. The size of squares and circles is proportional to the number of sequences displaying the same genotype. The open circles represent putative sequences in the evolutionary pathway. The solid lines on a network represent mutational connections among unique genotypes with at least a 95% degree of confidence, whereas the dashed lines represent a more tenuous connection. Click here for file

Additional file 6 ***F*_ST _estimates of seven MLST genes, two fluoroquinolone resistance genes, and *porB *gene (PIB)**. Sequences were analyzed together and partitioned by year of isolation. *P *< 0.05 are considered statistically significant, but only significant associations after Benferroni correction are shown in bold with an asterisk. Click here for file

Additional file 7 **Population genetics estimates for the Shanghai gonococcal population in individual genes**. Estimates of genetic diversity (*θ*) and recombination (*r *and *C*) for each of the seven housekeeping genes (*fumC*, *gdh*, *glnA*, *gnd*, *pilA*, *pyrD*, *serC*), *gyrA *and *parC *(Fluoroquinolone resistance genes) and the *porB *gene (PIA and PIB). Click here for file
