# Supplementary material for: Population dynamics of Neisseria gonorrhoeae in Shanghai, China: a comparative study
Source: BMC Infect Dis. 2010 Jan 21;10:13. doi: 10.1186/1471-2334-10-13 (PMC2822776; doi:10.1186/1471-2334-10-13)
Supplement: Additional file 1 — Epidemiological information for the isolates under study. All isolates were collected from patients seen in clinics of the Shanghai Skin Diseases Hospital. [file 1471-2334-10-13-S1.PDF]

Additional file 1, Table S1
Title: Epidemiological information for the isolates under study.
Description: All isolates were collected from patients seen in clinics of the Shanghai Skin Diseases Hospital.

Isolate	Collection date	Patient's gender	Patient's age	Geographic isolation
G786	04/2005	Male	48	Shanghai
G787	04/2005	Male	42	Shanghai
G788	04/2005	Male	27	Shanghai
G789	04/2005	Male	38	Shanghai
G790	04/2005	Male	72	Shanghai
G791	05/2005	Male	21	Shanghai
G792	05/2005	Male	50	Shanghai
G793	05/2005	Male	28	Shanghai
G794	05/2005	Male	24	Shanghai
G795	05/2005	Male	37	Shanghai
G796	06/2005	Male	38	Shanghai
G797	06/2005	Male	35	Shanghai
G798	07/2005	Male	31	Shanghai
G799	07/2005	Male	23	Shanghai
G800	07/2005	Male	24	Shanghai
G801	07/2005	Male	19	Shanghai
G802	08/2005	Male	18	Shanghai
G803	08/2005	Male	52	Shanghai
G804	10/2005	Male	44	Shanghai
G805	02/2001	Male	36	Shanghai
G806	02/2001	Male	42	Shanghai
G807	02/2001	Male	50	Shanghai
G808	02/2001	Male	28	Shanghai
G809	02/2002	Male	47	Shanghai
G810	02/2003	Male	22	Shanghai
G811	02/2003	Male	34	Shanghai
G812	02/2003	Male	31	Shanghai
G813	02/2003	Male	35	Shanghai
G814	02/2003	Male	39	Shanghai
G815	02/2003	Male	35	Shanghai
G816	02/2003	Male	59	Shanghai
G817	02/2003	Male	46	Shanghai
G818	03/2002	Male	31	Shanghai
G819	03/2002	Male	30	Shanghai
G820	03/2002	Male	40	Shanghai
G821	03/2002	Male	29	Shanghai
G822	03/2002	Male	53	Shanghai
G823	03/2002	Male	39	Shanghai
G824	03/2002	Male	48	Shanghai
G825	03/2002	Male	46	Shanghai
G826	03/2002	Male	38	Shanghai
G827	03/2003	Male	40	Shanghai
G828	03/2002	Male	29	Shanghai
G829	03/2002	Male	30	Shanghai
G830	03/2002	Male	40	Shanghai
G831	03/2002	Male	44	Shanghai
G832	04/2002	Male	41	Shanghai
G833	04/2002	Male	46	Shanghai
G834	04/2002	Male	42	Shanghai
G835	04/2002	Male	24	Shanghai
G836	03/2003	Male	34	Shanghai
G837	03/2003	Male	40	Shanghai
G838	03/2003	Male	46	Shanghai

Isolate	Collection date	Patient's gender	Patient's age	Geographic isolation
G839	03/2003	Male	33	Shanghai
G840	03/2003	Male	20	Shanghai
G841	03/2003	Male	38	Shanghai
G842	03/2003	Male	18	Shanghai
G843	03/2004	Male	27	Shanghai
G844	04/2003	Male	30	Shanghai
G845	04/2003	Male	25	Shanghai
G846	04/2003	Male	50	Shanghai
G847	04/2003	Male	31	Shanghai
G848	03/2004	Male	44	Shanghai
G849	03/2004	Male	31	Shanghai
G850	03/2004	Male	29	Shanghai
G851	03/2004	Male	28	Shanghai
G852	03/2004	Male	26	Shanghai
G853	03/2004	Male	27	Shanghai
G854	03/2004	Male	34	Shanghai
G855	03/2004	Male	42	Shanghai
G856	03/2004	Male	48	Shanghai
G857	03/2004	Male	40	Shanghai
G858	04/2004	Male	18	Shanghai
G859	04/2004	Male	37	Shanghai
G860	04/2004	Male	26	Shanghai
G861	04/2004	Male	40	Shanghai
G862	04/2004	Male	32	Shanghai
G863	04/2004	Male	31	Shanghai
G864	04/2004	Male	53	Shanghai
G865	04/2004	Male	28	Shanghai
G866	04/2004	Male	42	Shanghai
G867	09/2001	Male	26	Shanghai
G868	09/2001	Male	19	Shanghai
G869	09/2001	Male	41	Shanghai
G870	09/2001	Male	43	Shanghai
G871	09/2001	Male	34	Shanghai
G872	08/2001	Male	31	Shanghai
G873	08/2001	Male	28	Shanghai
G874	08/2001	Male	21	Shanghai
G875	08/2001	Male	48	Shanghai
G876	08/2001	Male	41	Shanghai
G877	08/2001	Male	23	Shanghai
G878	08/2001	Male	35	Shanghai
G879	08/2001	Male	37	Shanghai
G880	08/2001	Male	22	Shanghai
G881	11/2001	Male	45	Shanghai
