# Supplementary figures and images for: Population dynamics of Neisseria gonorrhoeae in Shanghai, China: a comparative study
Source: BMC Infect Dis. 2010 Jan 21;10:13. doi: 10.1186/1471-2334-10-13 (PMC2822776; doi:10.1186/1471-2334-10-13)

(A)

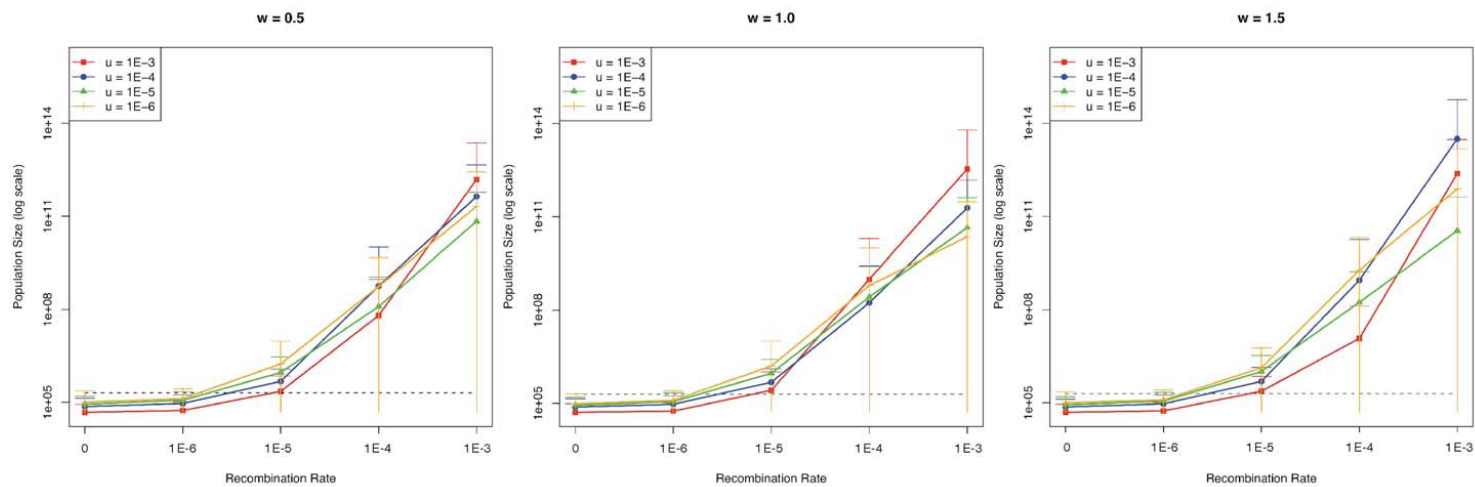

(B)

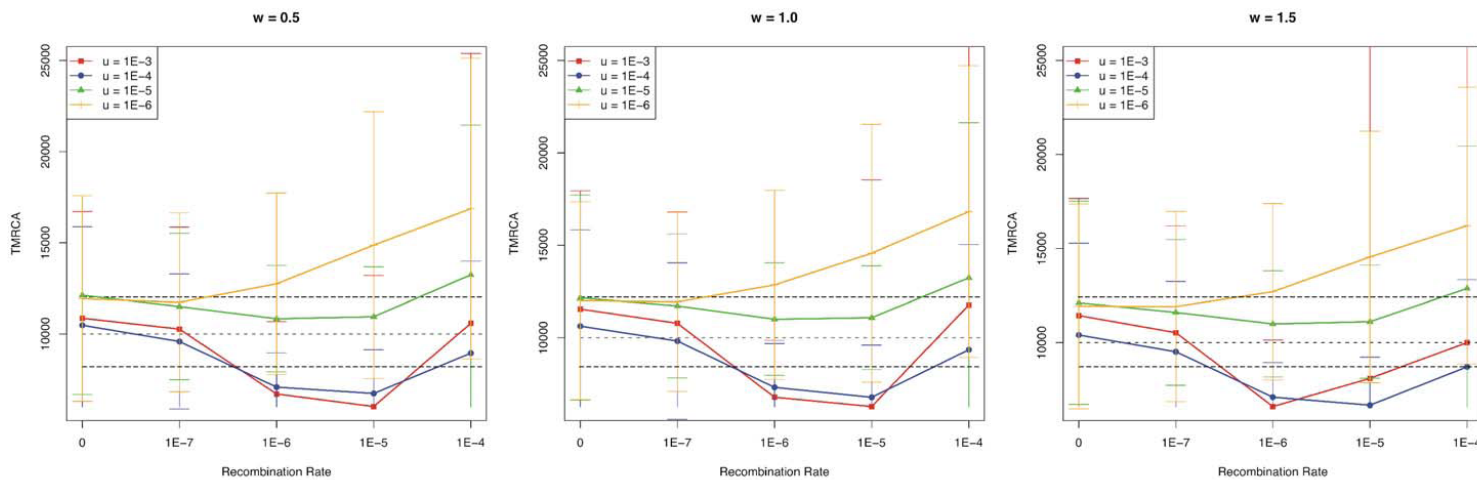

Supplement: Additional file 2 — BEAST simulations for population size (A) and TMRCA (B). Simulations performed under different values of recombination per site (X axis). 6,000 datasets were simulated in Recodon [59] under conditions reflecting average estimates for selection (ω), recombination (r) and substitution rate (μ) parameters in gonorrhea studies for the housekeeping, fluoroquinolone resistant, and porB genes. Simulated data were then analyzed in BEAST to assess the impact of recombination alone (ω = 1) and combined with adaptive selection (ω > 1) and purifying selection (ω < 1). [file 1471-2334-10-13-S2.PDF]

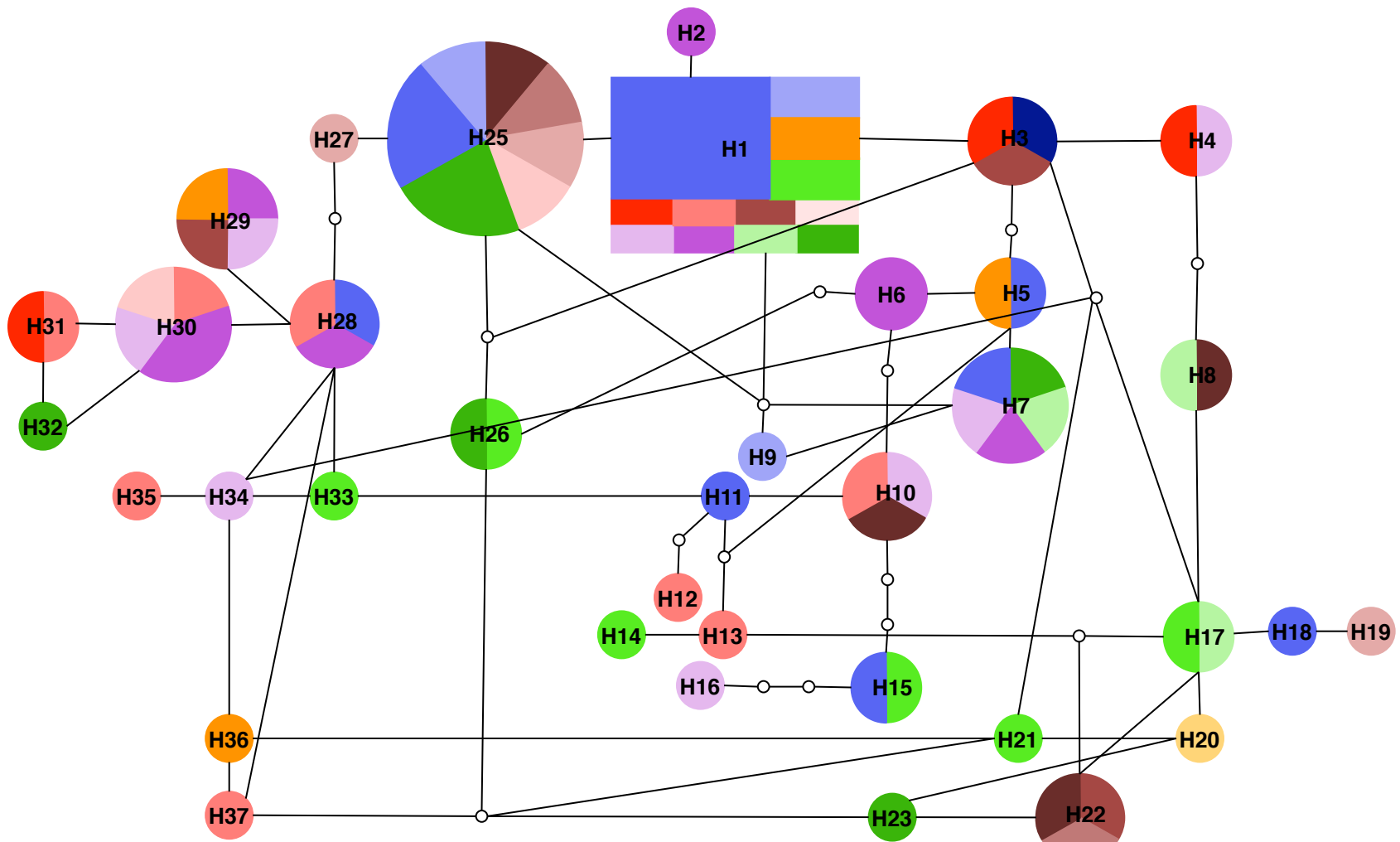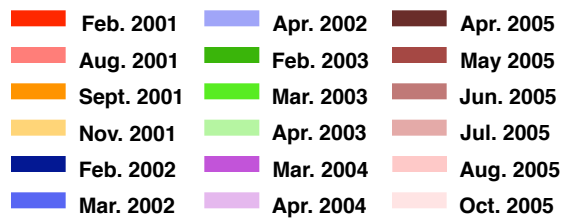

Supplement: Additional file 3 — Statistical parsimony network of concatenated fluoroquinolone resistance genes (parC and gyrA). The solid squares (putative outgroups) and the solid circles represent actual sequences derived from the strains analyzed. The size of squares and circles is proportional to the number of sequences displaying the same genotype. The open circles represent putative sequences in the evolutionary pathway. The solid lines on a network represent mutational connections among unique genotypes with at least a 95% degree of confidence, whereas the dashed lines represent a more tenuous connection. [file 1471-2334-10-13-S3.PDF]

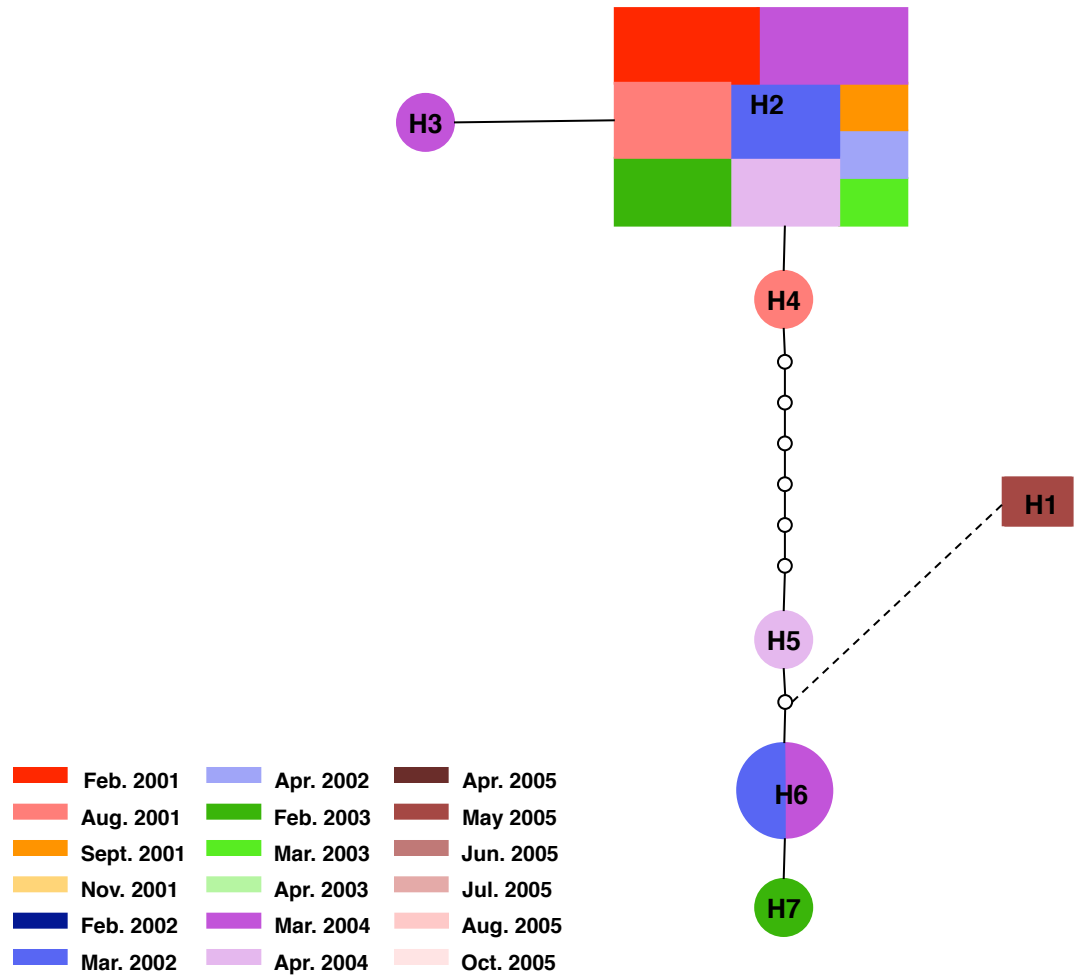

Supplement: Additional file 4 — Statistical parsimony network of porB gene (PIA). The solid squares (putative outgroups) and the solid circles represent actual sequences derived from the strains analyzed. The size of squares and circles is proportional to the number of sequences displaying the same genotype. The open circles represent putative sequences in the evolutionary pathway. The solid lines on a network represent mutational connections among unique genotypes with at least a 95% degree of confidence, whereas the dashed lines represent a more tenuous connection. [file 1471-2334-10-13-S4.PDF]

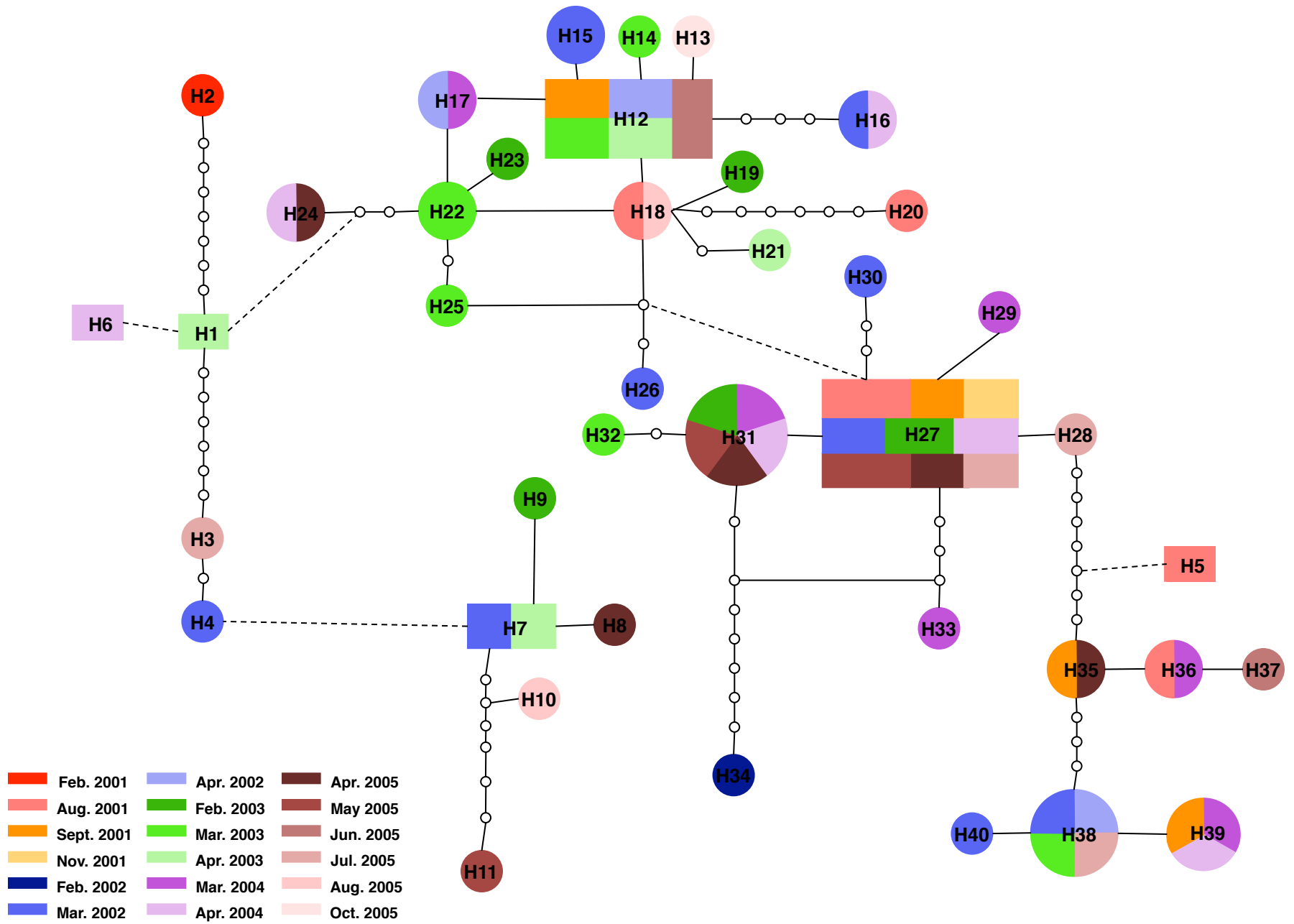

Supplement: Additional file 5 — Statistical parsimony network of porB gene (PIB). The solid squares (putative outgroups) and the solid circles represent actual sequences derived from the strains analyzed. The size of squares and circles is proportional to the number of sequences displaying the same genotype. The open circles represent putative sequences in the evolutionary pathway. The solid lines on a network represent mutational connections among unique genotypes with at least a 95% degree of confidence, whereas the dashed lines represent a more tenuous connection. [file 1471-2334-10-13-S5.PDF]
