# Supplementary material for: Population dynamics of Neisseria gonorrhoeae in Shanghai, China: a comparative study
Source: BMC Infect Dis. 2010 Jan 21;10:13. doi: 10.1186/1471-2334-10-13 (PMC2822776; doi:10.1186/1471-2334-10-13)
Supplement: Additional file 6 — FST estimates of seven MLST genes, two fluoroquinolone resistance genes, and porB gene (PIB). Sequences were analyzed together and partitioned by year of isolation. P < 0.05 are considered statistically significant, but only significant associations after Benferroni correction are shown in bold with an asterisk. [file 1471-2334-10-13-S6.PDF]

Additional file 2, Table S2
Title: F_{ST} estimates of seven MLST genes, two fluoroquinolone resistance genes, and *porB* gene (PIB).
Description: Sequences were analyzed together and partitioned by year of isolation. $P < 0.05$ are considered statistically significant, but only significant associations after Benferroni correction are shown in bold with an asterisk.

Loci/Partitions	F_{ST}	*P* values
MLST genes		
2001-2002-2003-2004-2005	0.001	0.438
2001-2002-2003-2004	0.007	0.358
2001-2002-2003-2005	0.002	0.413
2001-2002-2004-2005	0.005	0.416
2001-2003-2004-2005	-0.011	0.585
2002-2003-2004-2005	0.005	0.431
2001-2002-2003	0.001	0.387
2001-2002-2004	0.014	0.309
2001-2002-2005	0.01	0.317
2001-2003-2004	-0.004	0.466
2001-2003-2005	-0.01	0.563
2001-2004-2005	-0.014	0.644
2002-2003-2004	0.016	0.265
2002-2003-2005	0.006	0.389
2002-2004-2005	0.012	0.39
2003-2004-2005	-0.016	0.664
2001-2002	0.007	0.316
2001-2003	-0.008	0.525
2001-2004	-0.006	0.506
2001-2005	-0.005	0.484
2002-2003	0.005	0.337
2002-2004	0.04	0.094
2002-2005	0.029	0.15
2003-2004	0.002	0.376
2003-2005	-0.017	0.688
2004-2005	-0.032	0.933
gyrA and *parC*		
2001-2002-2003-2004-2005	0.048	0.193
2001-2002-2003-2004	0.043	0.227
2001-2002-2003-2005	0.061	0.117
2001-2002-2004-2005	0.069	0.166
2001-2003-2004-2005	0.018	0.289
2002-2003-2004-2005	0.05	0.152
2001-2002-2003	0.056	0.108
2001-2002-2004	0.065	0.263
2001-2002-2005	0.103	0.016
2001-2003-2004	0.004	0.396
2001-2003-2005	0.031	0.184
2001-2004-2005	0.024	0.318
2002-2003-2004	0.048	0.135
2002-2003-2005	0.054	0.157
2002-2004-2005	0.085	0.061
2003-2004-2005	0.015	0.253
2001-2002	0.116	0.01
2001-2003	0.022	0.174
2001-2004	-0.023	0.772
2001-2005	0.067	0.03
2002-2003	0.03	0.142

Loci/Partitions	F_{ST}	*P* values
gyrA and *parC*		
2002-2004	0.102	0.013
2002-2005	0.125	**0.007***
2003-2004	0.013	0.254
2003-2005	0.005	0.337
2004-2005	0.027	0.154
porB (PIB)		
2001-2002-2003-2004-2005	-0.003	0.481
2001-2002-2003-2004	-0.003	0.503
2001-2002-2003-2005	-0.001	0.452
2001-2002-2004-2005	-0.024	0.618
2001-2003-2004-2005	0.007	0.424
2002-2003-2004-2005	0.007	0.403
2001-2002-2003	-0.004	0.495
2001-2002-2004	-0.034	0.704
2001-2002-2005	-0.017	0.563
2001-2003-2004	0.016	0.407
2001-2003-2005	0.018	0.308
2001-2004-2005	-0.035	0.71
2002-2003-2004	0.011	0.41
2002-2003-2005	-0.002	0.446
2002-2004-2005	-0.011	0.489
2003-2004-2005	0.029	0.275
2001-2002	-0.025	0.648
2001-2003	0.044	0.149
2001-2004	-0.069	0.999
2001-2005	-0.02	0.581
2002-2003	-0.031	0.693
2002-2004	-0.007	0.466
2002-2005	-0.007	0.457
2003-2004	0.072	0.08
2003-2005	0.032	0.189
2004-2005	-0.017	0.561
