# Supplementary material for: Population dynamics of Neisseria gonorrhoeae in Shanghai, China: a comparative study
Source: BMC Infect Dis. 2010 Jan 21;10:13. doi: 10.1186/1471-2334-10-13 (PMC2822776; doi:10.1186/1471-2334-10-13)
Supplement: Additional file 7 — Population genetics estimates for the Shanghai gonococcal population in individual genes. Estimates of genetic diversity (θ) and recombination (r and C) for each of the seven housekeeping genes (fumC, gdh, glnA, gnd, pilA, pyrD, serC), gyrA and parC (Fluoroquinolone resistance genes) and the porB gene (PIA and PIB). [file 1471-2334-10-13-S7.PDF]

Additional file 3, Table S3

Title: Population genetics estimates for the Shanghai gonococcal population in individual genes.

Description: Estimates of genetic diversity (θ) and recombination (r and C) for each of the seven housekeeping genes (*fumC*, *gdh*, *glnA*, *gnd, pilA, pyrD*, *serC*), *gyrA* and *parC* (Fluoroquinolone resistance genees) and the *porB* gene (PIA and PIB).

Locus	L	θ	r (c/μ)	C
fumC	465	0.0002 [0.0002 to 0.0003]	$1.6e^{-5}$ [$1.0e^{-10}$ to 3.2]	$3.2e^{-9}$ [$2.0e^{-14}$ to 0.001]
gdh	501	0.003 [0.002 to 0.004]	12.9 [9.4 to 17.2]	0.04 [0.02 to 0.07]
glnA	576	0.003 [0.002 to 0.003]	2.6 [1.8 to 3.7]	0.008 [0.004 to 0.01]
gnd	594	0.002 [0.002 to 0.003]	8.3 [5.6 to 11.7]	0.02 [0.01 to 0.03]
pilA	420	0.007 [0.006 to 0.009]	3.5 [2.8 to 4.4]	0.02 [0.02 to 0.04]
pyrD	684	0.0005 [0.0004 to 0.0007]	$3.3e^{-5}$ [$1.0e^{-10}$ to 1.9]	16.5 e^{-9} [$4.0e^{-14}$ to 0.001]
serC	567	0.005 [0.003 to 0.007]	0.6 [0.3 to 1.4]	0.003 [0.0009 to 0.01]
gyrA	423	0.005 [0.004 to 0.006]	0.3 [0.04 to 0.8]	0.001 [0.0002 to 0.005]
parC	465	0.007 [0.006 to 0.009]	3.8 [2.6 to 5.2]	0.03 [0.02 to 0.05]
PIA	474	0.007 [0.004 to 0.016]	0.049 [0.002 to 0.23]	0.0003 [0.00001 to 0.004]
PIB	543	0.047 [0.035 to 0.067]	0.51 [0.33 to 0.73]	0.024 [0.01 to 0.05]

NOTE -- LAMARC's confidence intervals (5% and 95%) around the estimates of each parameter are given between brackets.

L = Sequence length in each gene.
